# A Theoretical Model of Reproductive Coercion and Abuse and Legal Entrapment: Barriers to Health, Safety, and Well-being for Mothers and Children

**DOI:** 10.1007/s10896-025-01034-5

**Published:** 2025-12-11

**Authors:** Kathryn J. Spearman, Sonja Ayeb-Karlsson, Dominique Guillaume, Sheridan Miyamoto, Kamila A. Alexander, Jacquelyn Campbell

**Affiliations:** 1 Ross & Carol Nese College of Nursing & Child Maltreatment Solutions Network, Pennsylvania State University, Pennsylvania, USA; 2 Everyday Disasters and Violence Research Group Lead, Institute for Risk and Disaster Reduction (IRDR), University College London (UCL), London, UK; 3 Johns Hopkins University School of Nursing, Baltimore, USA

**Keywords:** Reproductive coercion and abuse, Legal entrapment, Sexual violence, Family court, Intimate partner violence

## Abstract

**Purpose:**

This purpose of this article is to encourage the adoption of a theoretical model that centers the ways in which experiences of reproductive coercion and abuse (RCA) intersect with legal entrapment, gendered immobility, and adverse health consequences. This framework integrates disparate bodies of scholarship that have been neglected in prior examinations of RCA in order to provide a heuristic tool for research, practice, and policy.

**Methods:**

The authors discuss the existing literature on RCA and propose a theoretical model informed by feminist and reproductive justice theories, embedded in a socio-ecological model highlighting structural and social determinants of health.

**Results:**

Reproductive coercion and abuse (RCA) is a form of violence against women that interferes with a woman’s reproductive autonomy and freedom, contributing to adverse health and economic consequences. In the context of RCA, barriers to health exist at the societal level, community level, and interpersonal level resulting in legal entrapment and gendered immobility.

**Conclusion:**

This multi-level theoretical model integrates disparate scholarly lines of inquiry around RCA, gendered immobility, legal entrapment, and can serve to move the science forward on RCA to promote the health and well-being of mothers and children.

## Introduction

Reproductive coercion and abuse (RCA) is a form of violence against women that interferes with a woman’s reproductive autonomy.^1^ Reproductive and parenting experiences are shaped and governed explicitly and informally by institutions and governments (Browner & Sargent, 2021). Gender-based norms, expectations, policies, and discrimination around reproductive issues may constrain the physical, social, and economic movement of women, which has been referred to as gendered immobility. Gendered immobility can also result from legal or systemic entrapment (Tolmie et al., 2024), or the ways in which policies and legal systems perpetuate power imbalances and restrict an individual’s agency, autonomy, and freedom of movement. These constraints are particularly notable for pregnant individuals given social norms and the political and legal landscapes that govern reproductive health and shared parenting. This landscape is frequently changing (West, 2024) and varies across jurisdictions.

While scholars have explored broad domains of RCA from a socio-ecological perspective (Graham et al., 2023; Coleman et al., 2023; Khan et al., 2025), a more nuanced approach that integrates RCA with gendered immobility, legal entrapment, and the resulting economic and health consequences is missing from the literature. Most of the literature on RCA focuses on individual health consequences. There has been less focus on the social, economic, and legal repercussions of RCA experiences (Komazec & Farmer, 2024) and the multi-etiological ways in which this is connected to health outcomes through the lifespan. Centering experiences of intimate-partner-perpetrated^2^ RCA tactics within a context of legal entrapment and gendered immobility is necessary to demonstrate how both perpetrator and State actions (or lack thereof) result in entrapment and erosion to the safety, autonomy, and well-being of women and children.

The purpose of this article is to propose a theoretical model connecting intimate-partner perpetrated RCA with legal entrapment, gendered immobility, and adverse health consequences: The Reproductive Coercion and Abuse and Legal Entrapment Theoretical Model (Fig. 1). This proposed theoretical model of RCA and legal entrapment is informed by key theoretical frameworks, including feminist and reproductive justice theories (Roberts, 2017) and intersectionality (Crenshaw, 1991). Additionally, the model is embedded in a socio-ecological model (Heise, 1998; Coleman et al., 2023) highlighting structural (Solar & Irwin, 2010) and social determinants of health (Healthy People, 2030) – the factors that influence the conditions of daily life in which people live, learn, work, play, and age.

In the following sections, we review the relevant literature related to RCA, legal entrapment, and gendered immobility and integrate these disparate bodies of scholarship to elucidate the components of our theoretical model. We then discuss the ways in which societal (structural determinants of health), community (social determinants of health), and interpersonal level (intersectional positionality and RCA experiences) factors overlap and intersect to influence legal entrapment, gendered immobility, with downstream impacts to health outcomes (Fig. 1). Finally, we discuss the literature on identified health outcomes associated with RCA and provide recommendations for research, practice, and policy.

Figure 1 presents multi-level hypothesized relationships within the Reproductive Coercion and Abuse and Legal Entrapment Theoretical Model. Solid arrows indicate directional pathways, linking structural determinants of health to individual health trajectories through intermediary mechanisms. Solid arrows also link experiences of RCA at the interpersonal level to maternal child health consequences. Dotted arrows depict hypothesized associations among RCA, legal entrapment, and gendered immobility, with a recognition that RCA frequently co-occurs with IPV and coercive control. Adverse health consequences are exacerbated by experiences of legal entrapment and gendered immobility. Dotted lines represent potential moderators or stratifying variables, demonstrating how intersecting aspects of inequality could potentially exacerbate health and legal outcomes. Interpersonal level factors including RCA are more proximal to individual health consequences, but these are manifestations of structural and social inequalities, influenced by upstream conditions as well as intersectional positionality that shapes risk and vulnerability.

### Reproductive Coercion and Abuse

Reproductive coercion was first defined in 2010 (see Miller et al., 2010), and the literature and understanding of this phenomenon and its implications for health is still in its relatively nascent stages (Tarzia & Hegarty, 2021; Grace & Fleming, 2016). RCA is perpetrated at the interpersonal level between intimate partners but enabled by structural contexts that create vulnerability. RCA includes a constellation of behaviors aimed at controlling their female partner’s reproductive autonomy. RCA behaviors can include birth control sabotage; prevention of or forced abortion; medical control; physical, sexual, physiological, psychological, legal, and economic abuse (Hahn et al., 2025). These RCA behaviors may lead to pregnancy (“pregnancy inducing” or “pregnancy promoting”) (McCauley et al., 2017) or prevent a pregnancy from occurring or ending a pregnancy (“pregnancy harming” or “pregnancy preventing”). RCA frequently co-occurs with intimate terrorism (Johnson, 2010), a form of intimate partner violence (IPV) embedded in patterns of coercive and controlling tactics (Grace & Anderson, 2018; Stark, 2007) (Fig. 1).

RCA can occur throughout conception and prenatal periods, with the scientific discourse around RCA often ending with pregnancy. A broader perspective around RCA during the perinatal period and its intersection with other forms of interpersonal and institutional violence is necessary to improve maternal and infant morbidity and mortality, and to promote understanding of how RCA experiences may influence health outcomes throughout the life course and transmit intergenerationally to children. There is a need to understand the range of RCA behaviors, its intersection with coercive control, and the resulting constraints on women’s autonomy from co-parents or partners (Wood et al., 2022) from conception, childbirth, and post-partum and beyond. Supplemental Table 1 provides hypothetical examples of RCA behaviors and legal entrapment to provide nuanced illustrations of how RCA could potentially play out in healthcare, economic, and legal contexts. RCA experiences should be understood as a way for male partners or co-parents to control a woman’s reproductive resources, but also as a tactic to control and exploit their female partner’s economic and other resources as well.

RCA experiences need to be examined using an intersectional lens, and understood as context and culturally relevant. This includes culturally specific forms of RCA (Rahill et al., 2020) and the social and legal safety strategies that are available to survivors (Chavis & Hill, 2008). This can include the availability of abortion or an individual’s legal status in their place of residence. Defining the continuum of RCA in the context of legal entrapment has new importance in the aftermath of the US Supreme Court’s decision in *Dobbs v Jackson Women’s Health Organization* in the US and individual US states that are rolling back protections on women’s access to reproductive health care (Stoever, 2023; Hahn et al., 2025). In the wake of this decision a number of state legislatures have not only prohibited abortion but have introduced criminal statutes that punish women for fetal harm and miscarriages (Weigel, Sobel, & Salginicoff, 2020). Once an infant is born, survivors of RCA that do not remain in relationships with the person who impregnated them must continue to navigate co-parenting and family court legal systems, civil legal systems that require financial and economic resources to meaningfully access safety or justice.

In the US, Black women are overrepresented among survivors of RCA (Holiday et al., 2017), IPV (Stockman et al., 2015), and experience disproportionate rates of intimate partner homicide, particularly during pregnancy and the post-partum period (Wallace et al., 2021). Black and Indigenous women also experience disproportionate rates of State violence in the context of IPV – particularly around issues of State’s removing their children from their care and custody and incarceration (Roberts, 2017, 2022). Women with disabilities experience greater rates of RCA and IPV in addition to experiences such as forced sterilization that limit their reproductive autonomy (Wu et al., 2017; Serrato et al., 2021). Women with precarious legal status (Di Matteo & Scaramuzzino, 2022) are especially vulnerable to RCA at the intersection of both perpetrator and State violence, and may fear deportation and separation from their children should they seek legal recourse to address experiences of RCA and its consequences. These experiences of both perpetrator violence and State violence create compounding vulnerabilities, and lead to widening health inequities.

### Legal Entrapment

Placing experiences of RCA within a framework of legal entrapment is necessary to highlight the intersectional aspect of inequities associated with race, ethnicity, class, sex, gender, sexual identity, nationality, migration status, religion and other forms of oppression (Crenshaw, K.; 1991; Tolmie, Smith, & Wilson, 2024). This is especially necessary for the most marginalized of survivors, those who live at the intersection of partner/co-parent violence and structural violence. These experiences relate to both their experiences of violence and the safety strategies available to them within the context of entrapment (Bagwell-Gray et al., 2021). Entrapment is best understood as a “condition of unfreedom” (Stark & Hester, 2019). Gendered entrapment, as conceptualized by Stark (2007), occurs when personal liberties are constricted and controlled at the intersection of perpetrator tactics of coercion and structural conditions, like laws, policies, and society-level norms, that compel obedience indirectly. This results in restricted agency. Agency is central to understanding sexual and reproductive health outcomes, and discussions of individual agency often mask structural inequities that lead to diminished or inaccessible pathways to exercise autonomy in one’s own life.

Reproductive coercion, IPV, and other forms of coercive control must be centered within a broader framework of gendered entrapment. That is, survivors’ agency and autonomy is constrained by perpetrator behaviors and broader systemic patterns of harm perpetrated by states and institutions (Tolmie, Smith, & Wilson, 2024; Roberts, 2017). Legal systems worldwide are complicit in enforcing and maintaining male dominance over female sexual activity, reproduction, and parenting (Suk, 2023) and the ability for mothers to acquire economic capital (World Bank, 2024), particularly for the most marginalized women worldwide. These restricted liberties that are as a result of laws and policies, and how they are implemented at community levels, should be conceptualized as legal entrapment.

Men and societies at large benefit from women’s invisible reproductive work: unpaid, uncompensated, and undervalued labor that remains essential to society’s continued survival (Suk, 2023). Women of color and other marginalized groups are even more likely to have their reproductive work not only undervalued but often thought of as contributing to societal problems based on racist and misogynist stereotypes that blame individuals for historical and structural problems. The role that men/fathers play as ‘gatekeepers’ and controllers over women’s sexual and reproductive health and autonomy over their own bodies and economic choices and opportunities is still poorly understood (Wood et al., 2022). And the ways that state legal policies further exacerbate limits on women’s autonomy in the context of RCA has also not been thoroughly investigated. This focus on the social and structural contexts is necessary to move the discourse from examining individual health consequences, to the broader way that experiences of entrapment, RCA, and state violence intersect with structural and social determinants of health and perpetuate inequities (Fig. 1).

Research and practice needs to shift its gaze to the unseen violence – the underlying economic, legal, and social conditions that set the terms for unjust enrichment and abuses of power from some social groups at the expenses of others (Suk, 2023). This is particularly evident at the intersection of RCA and legal entrapment – how society is overentitled to the sacrifices and individual losses of freedom that women and mothers endure in relation to lack of control over their own reproductive and economic destinies. State control of reproduction and parenting often mirrors coercive tactics of perpetrators but is typically not conceptualized on the continuum of coercive control and may be viewed as a facilitator of coercive control (Tarzia & Hegarty, 2021; Tarzia & McKenzie, 2024). Legal entrapment is especially evident in laws in US states including Texas, Arizona, Arkansas and Missouri that currently (2025) state in statute that pregnant women cannot get divorced (American Pregnancy Association, n.d.).

There is scarce comprehensive data tracking how legal contexts constrain women’s economic opportunities once they are impregnated. However, anecdotal stories on family court judicial rulings, particularly in the US context, highlight the ways in which the economic mobility of pregnant women can be limited. For example, news media have reported on judicial rulings that it is reasonable to require a pregnant woman to obtain permission from the male who impregnated her to move out of state to pursue educational or economic opportunities, with a family court judge calling the pregnant mother’s “appropriation of the child while in utero was irresponsible, reprehensible” when she moved to attend university (Eckholm, 2013). Discourses around reproductive coercion and abuse must factor in the structural context of laws and policies that leaded to gendered immobility, e.g. that constrain women’s autonomy, their ability to seek educational and economic opportunities, and to provide for and nourish their children.

### Gendered Immobility

Less attention has been paid in research and practice to the role of gendered entrapment and immobility in the ways it intersects in the lives of pregnant women and affects health. While entrapment has been identified as a central feature of RCA (Bagwell-Gray et al., 2021), the concept of gendered entrapment and immobility imposed by courts, legal systems, and policy related to RCA and its impact to health has received little scholarly investigation. There is a need for increased understanding of the connections between health and involuntary (gendered) (im)mobility, geographical and psychological entrapment or “trapped” populations.

In the area of human (im)mobility and migration studies, research has been conducted into the diverse ways that feeling legally, socially, emotionally and psychologically trapped (based on the extended conceptualization of ‘trapped populations’) impact peoples’ (often women’s and children’s) mental health and well-being through ‘gendered entrapment and immobility’ (Harasym et al., 2022; Ayeb-Karlsson, 2020). This includes the ways that imposed gendered immobility increases the risk of being exposed to gender-based violence including sexual violence and child sexual abuse in disaster and humanitarian contexts (Ayeb-Karlsson, 2020). Even though the current literature body on “trapped populations” and gendered (im)mobility primarily has grown out of human geography and population studies (e.g. Ayeb-Karlsson et al., 2018, 2020), it has heavily borrowed from clinical psychology in terms of “feeling trapped” and how this overlaps with states of “depressiveness”, “hopelessness” and “helplessness.”

Extending (im)mobility decision-making and well-being is valuable for the conceptualization of RCA and legal entrapment. Displaced and refugee women have severe restrictions on their autonomy in the context of RCA as they may not be able to access legal recourse (Khan et al., 2025). Stressors and pressures, whether financial, political, environmental, legal, or health-related, such as in the context of the COVID-19 pandemic or in armed conflict or other humanitarian disasters, may intensify and increase already existing coercive and controlling behaviors, RCA, and IPV. Thereby, increasing the gendered immobility and entrapment that victim-survivors experience. These experiences of entrapment and immobility – constrained space for action (Stark, 2007) - are intertwined with structural and social determinants of health.

### Structural and Social Determinants of Health

Structural determinants of health at the societal level include gendered notions of caregiving and unpaid domestic labor, push/pull migration factors, immigration policies and citizenship (such as the ability to confer citizenship status to one’s child), and (*de jure*) family law practices and norms around shared custody and the provision of child support. These have further downstream impacts on social determinants of health (Fig. 1). These societal level factors intersect with experiences of RCA with individual positionality at the interpersonal level, and can exacerbate risks to health. Intersectional positionality refers to how an individual’s identities shape their experiences of vulnerability and privilege both within intimate relationships and in relation to institutional structures (Crenshaw, 1991).

#### Gendered notions of caregiving and domestic labor

These norms often guide how resources and opportunities are shared within families. The unequal provision of capital, resources, and opportunities – that disproportionately affects women, and in particular mothers – leads to conditions that can foster abuse of power. Such power imbalances are reinforced by discriminatory social institutions that impede women’s economic and social wellbeing, thus increasing the risk for violence and RCA.

#### De jure family law practices

Governments at local, state, federal, and tribal levels set out specific *de jure* (on the books) policies governing family and reproduction. These laws include laws that regulate reproduction, availability of family planning and contraception, and parenthood, marriage and divorce. Equal rights for women as compared to men in marriage and divorce are critical for women’s autonomy, agency, economic security, and safety (World Bank, 2024). Policies such as the statutory presumption of contact with both parents as in the child’s best interest can create vulnerabilities for survivors of RCA. Young girls and adolescents are particularly vulnerable to RCA. Child marriage can be the result of RCA, or RCA may be part of a marriage where the female is under 18. As an illustration of how this results in legal entrapment, for example, in the US, in the 38 states that permit child marriage, girls who are married before the age of 18 are unable to legally obtain a divorce in the US until they are over the age of 18 (Schuman, 2018). Other family laws particularly relevant to the idea of RCA and legal entrapment are those that address domestic violence – currently 86 countries do not have statutory laws on domestic violence or if it is addressed, the laws are insufficient (World Bank, 2024). In patrilineal societies, customary law may typically confer a presumption of custody to fathers (Raday, 2019).

#### Immigration policies and push-pull migration factors

Immigration policies are also an important societal level factor that contribute to risk for legal entrapment. Virtually worldwide, citizenship status is automatically conferred through patrilineage, but in 28 countries around the world, mothers are prohibited from conferring their citizenship status to their children (World Bank, 2024). Most countries (currently 103 countries) are signatories to The Hague Convention, meaning that in case a child is removed from its habitual residence by a mother fleeing domestic violence (back home or to another country) without the permission of the perpetrator father or the family court the child will swiftly be returned to its jurisdiction. Re-entry to the father’s country comes with punitive risks, often resulting in child removal or even the mother’s imprisonment.

RCA and other forms of IPV and inadequate legal responses that lack pathways to protection for women and girls who are victims of violence have been implicated as push factors. Push factors refer to unfavorable aspects about a home country that can inform women’s decision to migrate from their home country to other countries. This phenomenon has become increasingly apparent with a rising number of women and girls fleeing from Latin America to the U.S., largely driven by violence and economic insecurity (Parish, 2017). In addition to RCA being a cause of migration, women also have a substantially increased risk of experiencing sexual violence during their migration route (Barbara et al., 2017; Tan & Kuschminder, 2022). Estimates from Amnesty International indicate between 60 and 80% of female migrants traveling through Mexico to the U.S. are raped along the way (Parish, 2017). Similar reports have been documented in Europe in which refugees escaping Syria and Iraq report experiencing physical abuse and forced sex by smugglers, security staff, and other refugees (Amnesty International, 2016). These acts are so commonplace that studies have reported women take contraceptives to prevent pregnancies prior to migrating; thus, indicating that women are cognizant of the risks posed to them during the migration journey and actively resist that entrapment. Although support services may be readily available within their destination country, migrant women who are victims of RCA may face numerous challenges in accessing sexual and reproductive health (SRH) information and care services due to language barriers, difficulty navigating the health system, lack of support, cultural barriers, and undocumented status. Despite this, women continue to undertake the risk of migrating to countries offering pull factors or favorable aspects of a country. Pull factors can include legal protections from violence and the possibility of asylum (Letona et al., 2023; Khouani et al., 2023). Countries with more pull factors, particularly countries that have lower discriminatory social institutions and that promote greater working opportunities for women can significantly influence women’s decisions to migrate. Conversely, gender norms that may reinforce inequalities can also constrain women’s ability to migrate. For example, a woman who is financially dependent on her husband and has a lack of resources and support may be less likely to have the opportunity to migrate to pursue educational or vocational opportunities. This denotes the tremendous impact that gender and social inequities that perpetuate RC and violence can have on women’s livelihoods.

### Community Level

#### De facto (in practice) family court responses

While *de jure* family laws in some jurisdictions may promote gender equality, how these laws are implemented and adopted in practice, or *de facto*, varies by local jurisdiction. Implementation gaps, breakdown in the rule of law, or institutional betrayal harms survivors of violence. These factors can lead to legal entrapment; such as family law judicial rulings that require women to continue to co-parent with the men who impregnated them through rape. Furthermore, deficiencies in child support enforcement and other child maintenance arrangements by states, contributes to gender-based poverty and gendered post-divorce financial disparities (Raday, 2019). Understanding how legal responses vary for women who experience RCA is an important consideration for future studies.

#### Community services and support

The ability to access and the availability of culturally safe and responsive services to help women who have experienced RCA may differ by geographical region (e.g., urban versus rural) and for individuals with different intersectional identities, including legal status. Additionally, the support of extended family and friends can be crucial to mitigate both the health and economic toll of RC experiences for mothers and their children; yet for migrant women and women with precarious legal status, they may have been further separated or isolated from support and may need additional legal and advocacy supports.

### Interpersonal Level

Interpersonal or “partner” or co-parent related factors include the perpetration of specific RCA behaviors (Supplemental Table 1), but also relative power, financial status, access to resources, history of violence, disparate immigration or documentation status, and other intersectional identities (Fig. 1) that may vary across jurisdictions. Perpetrators of RCA use women’s reproductive abilities as a “weapon against them” (Tarzia et al., 2019), which is further compounded in the ways that states regulate and control women’s reproductive capacities and communities respond to RCA, IPV, and parenting.

#### Economic consequences and RCA

Economic resources and stability are an important domain of social determinants of health (Fig. 1) and inextricably linked to health outcomes. Although the economic toll of RCA in the lives of women and through the lifespan has not yet been quantified, childbearing exacts an economic toll on mothers. Pregnancy and childbearing impacts mothers’ ability to participate in labor markets relative to men/fathers. In a recent study of gender wage gaps across 134 countries representing 95% of the world’s population, child penalties (loss of earnings after having a child), were evident in every country for women after the birth of their first child, although the magnitude of this difference varied dramatically from region to region (Kleven et al., 2023). A study by the World Bank (2024) indicated no country in the world offers women the same workforce opportunities as men, and deficiencies in safety (e.g., from gendered experiences of violence), childcare, and legal protections are leading contributors to preventing women from obtaining their potential in the workforce. In most of North America, Europe, and Australia, more women experience a child penalty compared to men. Parenthood is a non-economic event for men, and men’s wages typically rise when becoming fathers (Kleven et al., 2023). In Europe, mothers in Denmark experience the smallest child penalty of a 14% decline following the birth of their first child. For US mothers, becoming a mother reduces female earnings by 33% and reduces employment by 25%, as compared to fathers’ earnings which do not decline (Kleven, 2022).

Pregnancy often involves relationships that leave pregnant women and mothers economically dependent on others. This vulnerability is reinforced by States’ neglect of mothers and caregiver needs through inadequate policies, which should be viewed as a structural determinant of health. Caregiving responsibilities are valued far less than market work. Because of continued gendered stereotypes and expectations, this can inhibit women’s abilities to enjoy autonomy in life pursuits (Suk, 2023) and can result in further gendered immobility. These compounding factors result in diminished opportunities to acquire economic capital and protect and provide for themselves and their children (World Bank, 2024). The power imbalance that results between co-parents when women are unable to acquire economic capital at the same rates as their co-parents can further disadvantage them and lead to legal entrapment – particularly in regions of the world where access to family court systems requires financial resources (e.g., to obtain an attorney or experts). Additionally, economic abuse may co-occur along with RCA, IPV, and coercive control; yet economic abuse is often not explicitly defined in laws related to domestic violence. The opportunities for victims of RCA and economic abuse to obtain relief from legal systems is scant without improvements in written statutes. No studies to our knowledge have attempted to measure the economic consequences, lost productivity, and poverty resulting from RCA; yet the adverse health consequences that stem from experiences of reproductive coercion, childbearing, and child rearing do lead to an economic toll for mothers. Beyond the human toll, the cost of experiences of IPV and childhood adversity to GDP are measurable and have been well documented, although are likely underestimated (Peterson et al., 2023).

### Maternal-child Health Consequences of Reproductive Coercion and Abuse

RCA impacts health outcomes through multi-level pathways (Fig. 1). Interpersonal behaviors that encompass RCA such as forcing women to engage in sex without condoms and birth control sabotage can lead to numerous, well-documented clinical implications including sexually transmitted infections (STIs) and HIV, unintended pregnancy, miscarriage or abortion, poor pregnancy outcomes, immunological suppression due to stress, psychological trauma, and challenges to accessing prenatal, postpartum, and well child care (Wood et al., 2022; Grace & Anderson, 2018; Park et al., 2016). RCA experiences coupled with other forms of intimate terrorism may indicate that a woman is in danger of lethal violence (Bagwell-Gray et al., 2021).

#### STI risk

Multiple pathways have been illustrated between RCA and increased HIV and STI risk (O’Malley et al., 2021). RCA is a salient risk factor in the acquisition of HIV and other STIs due to the reduced likelihood of condom use, lower intentions of using or buying condoms, lack of advocacy and fear in discussing condom use with partners, coupled with additional barriers in negotiating condom use as a result of power-imbalanced relationships (Miller et al., 2010; Capasso et al., 2019; Anderson et al., 2017). In the context of HIV prevention, women who experience RCA are less likely to access pre-exposure prophylaxis (PrEP) and post-exposure prophylaxis (PEP) which are effective biomedical strategies in reducing HIV acquisition (Kim & Martin, 2023). Accessing PrEP and/or PEP may be a challenge among women experiencing RCA due to barriers imposed by co-parents resulting in victims experiencing difficulties in accessing health services (Kim & Martin, 2023). In addition to HIV, women who have experienced RCA have a notably elevated risk of being diagnosed with a laboratory-confirmed STI (Capasso et al., 2019; Grace et al., 2023; Hill et al., 2019; Kraft et al., 2021). Repeated or untreated STIs are a risk factor for infertility and other problematic reproductive health conditions such as interference with sexual pleasure. This STI risk is further exacerbated if women are simultaneously experiencing other forms of IPV.

### Unintended pregnancy and poor pregnancy outcomes

Formative literature has highlighted strong links between RCA and other forms of IPV with unintended pregnancies, defined as pregnancies that are unwanted, unplanned, or mistimed at the time of conception (Yazdkhasti, Pourreza, Pirak, & Abdi, 2015). The health consequences (e.g. mental distress, increased social stressors, increased risk of comorbidities, unsafe abortions) of unintended pregnancies among victims of RCA are of significant concern given that they can drastically lower women’s engagement in maternal and neonatal health services, thus potentially contributing to a downstream cascade of worsened overall maternal and child health outcomes. While not as readily studied, women who are victims of RCA have a substantially higher risk for rapid repeat pregnancies (Silverman et al., 2006) which have also been linked to adverse health consequences for women and their children.

#### Adverse fetal, infant, and child outcomes

Data indicates that infants born to mothers experiencing RCA have a higher likelihood of preterm birth and low infant birth weight which are key indicators of maternal health and are associated with higher infant mortality, delayed infant bonding and caretaking, child developmental and behavioral disorders (Fay & Yee, 2020; Liu et al., 2016). These health effects have lasting repercussions and long-term implications across the lifespan and intergenerational consequences.

#### Intimate partner homicide

Homicide is a leading cause of pregnancy-associated mortality. Limited research has examined the role that RCA experiences play as a risk factor or the potential correlation of homicides or deaths due to coerced suicides of pregnant women (Campbell et al., 2021; Smith et al., 2023). A strong predictor of IPV during pregnancy is a male partner not wanting the pregnancy, and wanting to stop abuse is a commonly cited reason by women seeking abortion (Chibber et al., 2014). A recent study by Wallace and colleagues (2024) found that rates of homicide increased for pregnant women following the enactment of policies that curtailed the availability of abortion. Moreover, among adolescent victims of intimate partner homicide, one of the most commonly listed reasons among law enforcement narratives was pregnancy (e.g., the male partner did not want the adolescent woman to have the child and killed her) (Wallace et al., 2024). Girls and adolescents under the age of 18 are also the age group with exposure to the most vulnerable and complicated legal landscape and fewest legal protections.

## Discussion

The aim of this article is to introduce a theoretical model of reproductive coercion and legal entrapment that can advance an understanding of this intersection in research, policy, and practice. Our theoretical model highlights how RCA is embedded in a socio-ecological context and how interpersonal-level RCA tactics intersect with legal entrapment (Supplemental Table 1), gendered immobility, and structural and social determinants of health (Fig. 1). This theoretical model expands upon and integrates existing scholarship to inform methodological inquiry into barriers to the safety, health, and well-being of maternal child dyads. This theoretical model can provide guidance for operationalizing variables and concepts related to RCA, legal entrapment, and gendered immobility. As a heuristic tool, this model has the potential to contribute to empirical, theoretical, and practice literature by integrating disparate bodies of literature to provide a more holistic overview of how RCA affects health. It can also help policy-makers identify potential areas for intervention; for example, improving statutory definitions of IPV to incorporate aspects of RCA and economic abuse or improving family law statutes to protect survivors and their children. Experiences of RCA and implications for adverse health consequences must be understood in the context of legal, economic, and social environments. Legal entrapment as a result of RCA contributes to adverse health consequences, increased morbidity and mortality, and thus warrants further attention by health, legal systems and in policy.

A health equity lens requires addressing factors influencing external locus of control, in particular the barriers including legal policies and legal actors (e.g. family court professionals) who may constrain the ability for women to obtain both health and economic opportunities for the benefit of themselves and their children. State policies and responses to violence within families and how reproduction and parenting are regulated may lead to legal entrapment for survivors, with States and ex-partners having more say over economic and health opportunities for women and children that the individual liberty and agency for women themselves. In some instances, policies may even unwittingly act as an incentive for abusive perpetrators to engage in RCA behaviors to use co-parenting as a tool of power and control over partners.

Even in healthy relationships, men and women must negotiate economic trade-offs, and negotiate decisions around reproduction and parenting. Childbearing motivations and decisions can be both joyful and fraught for both male and female partners, and discordance among desires around pregnancy, pregnancy timing, and parenthood can further complicate matters. Work by Alexander and colleagues (2021) identified that men may also feel ‘entrapped’ by the financial responsibilities (e.g., child support) of children conceived or that some male participants drew upon misogynist stereotypes, such as women’s “selfish” motives to entrap a male partner to obtain child support or government assistance. It is important to note that women may also use pregnancy as a way to try to keep their male partner in a relationship (Tarzia & Hegarty, 2021; Grace & Miller, 2023) – yet this doesn’t result in a woman’s ‘unwanted’ pregnancy. Ultimately it is a woman whose body bears the physical risks and consequences for bringing a child into the world, and suffers disproportionate economic consequences. Lack of access to abortion is associated with fatal violence for pregnant and post-partum women (Wallace et al., 2024). These disparities and risks for homicide are not noted when men become unwilling fathers.

Survivors of RCA are robbed of the ability to determine their own course and autonomy over their lives and their children. Not only because of financial and economic constraints that come with raising a child, but because of legal systems that impede the ability to pursue economic and educational opportunities that would improve standards of living and improve their access to positive social determinants of health as well. Patriarchal legal system norms reinforce women’s vulnerability and subjugate women’s autonomy. These legal orders are profound structural determinants of health. Moreover, legal orders that do not guarantee reasonable accommodations for pregnancy and motherhood implicitly assumes that society is entitled to women sacrificing their livelihoods and their lives to absorb the cost of bringing children into the world (Suk 2023).

### Recommendations for research, practice, and Policy

Future research should focus on developing measures of RCA and IPV in the perinatal period that incorporate items that assess for structural violence and entrapment related to legal policies and system responses. For example, this could manifest as wanting an abortion, but this not being available; being prohibited from breastfeeding via court order (Rathus et al., 2019); experiences of state violence such as being misidentified as primary aggressor and arrested, losing custody of children; experiences of legal abuse or being accused of ‘parental alienation’ in trying to protect children or obtain separation from an abusive co-parent. Precise measurement is important to further understandings of how RCA and coercive control may escalate over time, and how it may intersect with lethal danger. For example, further research on how perpetrators may attempt to punish a woman for not aborting a child by terrorizing her and child, including through the courts by using legal abuse to retaliate through seeking full custody for not obtaining abortion. Escalation of abuse during pregnancy signals dangerous perpetrators and is associated with risk for homicide; therefore, understanding these patterns are important for preventative efforts to reduce morbidity and mortality. With the exception of Willie et al. (2019), scholars have not examined the role of RCA and child development. Given that early environments exert a profound influence on human development and health through the lifespan (Shonkoff et al., 2012; Wal lack & Thornburg, 2016), research efforts should also focus on the impact of RCA behaviors on child development and child health outcomes.

Building on the framework proposed by Tarzia et al. (2019), research on RCA should distinguish between pregnancy-promoting RCA behaviors (McCauley et al., 2017), pregnancy-preventing or harming RCA behaviors (Tarzia et al., 2019) and legal entrapment (Supplemental Table 1). Future research should assess economic consequences to individual women as well as to society at large specific to RCA. Human suffering and disadvantage come with measurable material losses and economic consequences, and research should examine the way that RCA and legal entrapment impact domains of social determinants of health, particularly poverty and access to economic stability, and exacerbates health disparities. Research on RCA should explore the intersection with coercive control and chemical control, e.g., perpetrator’s control of other medications and substances that reduce the survivor’s capacity for autonomy and health (Walker et al., 2023) in the perinatal period. Future research also needs to explore women’s agency and resistance to RCA and subsequent legal entrapment with careful assessment of what promotes positive health outcomes for mothers and their children and mitigates negative sequelae. There needs to be research on healthy resolution of couple conflict in areas of reproductive decision making to give direction for future prevention interventions.

Health care systems should consider frameworks that assess barriers to health that involve legal entrapment and the ways the court decisions entrap and limit access to health care. Health care systems can promote the welfare of women, children, fathers and families by cultivating cultures of care, through advocating for societal and community-level policies and resources that may be protective. At an individual level, voicing concerns if health care professionals witness overbearing, demeaning, or controlling behaviors – particularly during pregnancy and childbirth - may help validate women’s experiences and help with future help-seeking (Buchanan & Humphreys, 2021). Health care professionals should provide universal education and assessment about IPV/RCA during reproductive health visits. Health care professionals can also offer women options for contraceptive options that are less likely to be manipulated by partners (McCauley et al., 2017). Furthermore, health care practitioners can connect patients with social work or legal services, and home visiting or nurse-legal partnerships may provide opportunities to intervene with pregnant women to provide civil legal remedies that can address social determinants of health (Tobin-Tyler & Teitelbaum, 2019).

Laws should protect the rights of women and children, not be complicit in or exacerbate the abuse they experience. Societal level factors that act as protective factors can include government policies that support access to abortion and other forms of long-acting contraception and reproductive health care, and legal norms and policies that support gender equality and promote women’s access to educational and economic opportunities. This can include policies that minimize the individual burden on women, such as access to affordable childcare and paid family leave (Tarzia et al., 2019). Worldwide, these policies continue to be inadequate to support mothers and their children (World Bank, 2024).

State legislators need to be educated on these issues so that they can craft laws that support prevention of RCA. In the US, state legislative bodies are only 33% female in 2023, and this is the highest per cent there has ever been. Yet 10 states have less than 24% female members (National Conference of State Legislatures, 2023). We need to continue to work for gender equity in our policy making bodies, our courts, in pay for traditional outside the home work, and in promoting family leave policies when infants are born. Many countries in Europe have worked hard for gender equity in the laws and policies and those same countries have lower rates of IPV, although RCA has not been measured in them. Family courts should consider evidence on RCA and IPV in custody and parenting time decisions. In family court systems, mothers are often negatively framed as ‘alienators’ or ‘hostile gatekeepers’ over father’s relationships with their children when raising safeguarding concerns due to domestic violence (Spearman et al., 2022; Austin et al., 2013). Other maternal actions, such as mothers’ attempts to continue to breastfeed their infants following separation from a co-parent may be characterized in family courts as a tactic to withhold the father’s access to the child, rather than as a decision that is health promotive and evidence-based in the child’s best interest (Rathus et al., 2019). Not all states have statutes that require family courts to consider breastfeeding when making decisions on the best interest of the child in custody and visitation decisions, but breastfeeding should be encouraged and supported because of the numerous health benefits and protective benefits conferred on nursing mothers and their infants (La Leche League, 2022).

The United Kingdom recently announced (October 2025) revisions to family law to eliminate the statutory presumption of contact with both parents as in the best interests of the child and also announced plans to automatically restrict parental responsibility for rapists whose crimes resulted in the birth of a child (Campbell & Crew, 2025). These types of policy changes have the potential to reduce legal entrapment and promote the health, safety, and well-being of mothers who have experienced RCA and for their children.

Furthermore, creating protective guardrails to limit the risk of future violence and coercion is of utmost importance. Family court decision makers should receive training on all forms of family violence, including RCA and other coercive tactics that extend beyond physical violence. Policies that promote economic security for women, flexible workplace policies, improved equality in legal and family law frameworks, and policies that promote access to affordable childcare can reduce the economic vulnerabilities created by RCA. Said another way, policies that limit individual women’s freedom and autonomy around reproductive choice, incentivize perpetrators to engage in RCA behaviors, because it creates another lever of power and control.

## Conclusion

We propose a theoretical model that integrates the literature on legal entrapment, gendered immobility, and reproductive coercion and abuse. Taking a socio-ecological perspective, particularly in the ways that State action or inaction can exacerbate abuse experienced at an individual level is important for research, practice, and policy. Through this broader lens of structural and social determinants of health, the science of RCA and protective policies can be moved forward, so that all mothers and children have the opportunities to live healthier lives.

## Supplementary Material

Spearman et al_Supplemental Table 1_RCA and legal entrapment

**Supplementary Information** The online version contains supplementary material available at https://doi.org/10.1007/s10896-025-01034-5.

## Figures and Tables

**Fig. 1:**
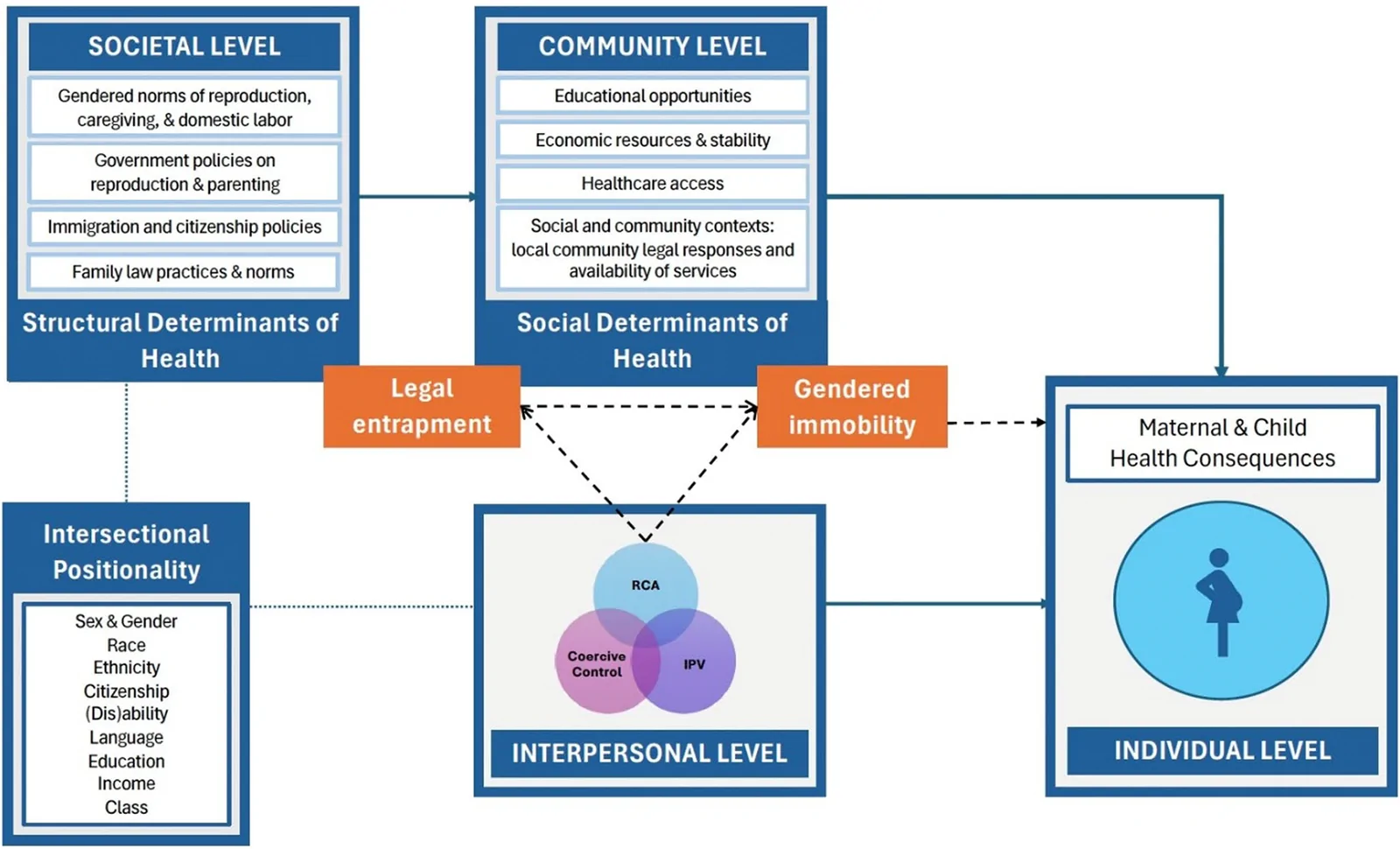
The reproductive coercion and abuse and legal entrapment theoretical model

## Data Availability

Not applicable.

## Footnotes

1 …

2 …
