# Supplementary material for: A Theoretical Model of Reproductive Coercion and Abuse and Legal Entrapment: Barriers to Health, Safety, and Well-being for Mothers and Children
Source: J Fam Violence. Author manuscript; Available in PMC 2026 Oct 17. (PMC13671256; doi:10.1007/s10896-025-01034-5)
Supplement: Supplementary file 1 [file NIHMS2209390-supplement-Spearman_et_al_Supplemental_Table_1_RCA_and_legal_entrapment.pdf]

Supplemental Table 1: Hypothetical examples to illustrate RCA behaviors and legal entrapment

RCA Behaviors	Pregnancy promoting	Pregnancy or maternal/infant harming	Legal system entrapment
**Physical**	Physical violence:<br>▪ to threaten or coerce pregnancy<br>▪ when a woman tries to use contraception<br>▪ to intimidate and induce fear and thereby control and coerce her longer-term	▪ Direct threats with firearms (e.g., holding a firearm to pregnant woman's head)<br>Physical violence to:<br>▪ induce miscarriage (such as throwing her down stairs)<br>▪ target fetus and abdominal area during pregnancy<br>▪ punish a woman for becoming pregnant<br>▪ punish a woman for not producing enough sons (Khan et al., 2025)	Legal system responses and norms that<br>▪ grant perpetrators of IPV primary custody of their children, making mothers dependent on abusive ex-partners/co-parents for access to their children<br>▪ confer patrilineage and custody to abusive fathers regardless of physical violence perpetrated against women and children<br>▪ criminalize non-offending mothers and remove their children from their care for "failure to protect"
**Physiological**	Coercing and controlling behaviors that include:<br>▪ control over rest and sleep, prenatal vitamins, control over certain food and drinks, or activities	▪ Withholding food<br>▪ Disrupting sleep<br>▪ Ignoring or withholding pregnancy needs, including prenatal vitamins<br>▪ Requiring hard labor, ignoring additional rest needs or other physiological needs<br>▪ Berating a woman during childbirth<br>▪ Requiring a woman adhere to certain behaviors during childbirth	▪ Forced sterilization (McKenzie et al., 2022)<br>▪ Regulation and criminalization of women for miscarriages
**Sexual**	▪ "Stealthing" – condom removal or condom sabotage<br>▪ Forced sex to cause pregnancy<br>▪ Ignoring woman's requests to use withdrawal method<br>▪ Transactional or survival sex<br>▪ Sexual assault once asleep or passed out to induce pregnancy	▪ Threatening or sharing intimate images ("revenge porn") or images during childbirth	▪ Legal system responses that requiring women to co-parent with their rapists
**Psychological**	▪ Emotional pressure to coerce a woman to become pregnant	▪ Emotional pressure to coerce a woman to end a pregnancy	▪ Using legal systems or power of state to isolate women or threaten women, such as through threats of loss of

	▪ Threats of harm if she does not get pregnant<br>▪ Threats of leaving relationship or getting with another partner if she does not get pregnant<br>▪ Emotional manipulation into planning a family and abandoning or threatening to abandon her and baby as she conceives	▪ Berating, blaming, or emotional abuse towards a woman for becoming pregnant or for not having a child of the desired sex (e.g., male offspring)<br>▪ Emotional disregard, spurning<br>▪ Threats of harm if she does not end pregnancy<br>▪ Threats of public humiliation and targeted reputational damage if she keeps the baby<br>▪ Threats of abandonment if she keeps baby<br>▪ Enforced isolation<br>▪ Other threats of harm with firearms, weapons<br>▪ Driving dangerously	custody or threats that they cannot interact with maternal support systems including extended family, limiting freedom of movement (going out of state)
**Economic**	▪ Threats to withdraw financial support unless she agrees to becoming pregnant and having a baby	▪ Threatening harm if a woman requests child support or other economic support for child<br>▪ Threats to withdraw financial support if she keeps baby<br>▪ Refusing to support pregnant woman and child financially<br>▪ Partner/co-parent's disinterest in pregnancy and infant to harm woman	▪ Using power of state to enforce and limit mobility or opportunity to pursue educational or economic opportunities<br>▪ States rendering harsh financial judgments against survivors<br>▪ Ordering survivors of IPV and RCA to pay child support to their rapists or abusers when they try to escape violence and are under resourced compared to perpetrators
**Legal**	▪ Forced marriage as a result of pregnancy<br>▪ Legal action to hinder abortion	▪ Prenup agreement containing amount of children or specific sex of child to be conceived as part of marriage	▪ Use of legal system to enact revenge for failure to end a pregnancy – such as seeking primary custody of child and are rewarded by legal system<br>▪ Use of legal system – such as immigration status to trap mothers and child(ren) – enhancing vulnerability such as leaving partner off immigration status documents<br>▪ Using legal system to to prosecute woman for seeking abortion in other jurisdiction
**Medical**	▪ Controlling access to health care environments to obtain contraception<br>▪ Withholding contraception	▪ Harassment or stalking in prenatal or antenatal health care setting<br>▪ Trying to prevent access or create impediments to	▪ Obtaining legal orders using paternity status to impede maternal medical care or infant health care

		prenatal and antenatal health care<br>▪ Refusal to support or attend prenatal or antenatal health care appointments<br>▪ Refusal to provide support for specialized medical needs<br>▪ Withholding insurance and insurance information for prenatal, antenatal, and infant care<br>▪ Sabotaging antenatal and prenatal health care (such as by withholding important genetic health details) or harassing her prior to or during appointments<br>▪ Displays of controlling and demeaning behaviors during antenatal appointments	
**Abortion**	▪ Refusing to allow her to obtain an abortion<br>▪ Sabotaging or manipulating her out of following through with abortion<br>▪ Exchanging or sabotaging prescribed medicine to induce abortion	▪ Trying to threaten, coerce, or otherwise force woman to get abortion against her wishes<br>▪ Spiking food or beverages with abortifacients to induce miscarriage	▪ Using legal system or legal abuse to prevent abortion<br>▪ State laws and policies that prohibit abortion
**Birth control sabotage**	▪ Sabotaging contraception<br>▪ Condom removal or intentional covert damage	▪ Spiking food/beverages with contraceptives to prevent pregnancy	▪ State laws and policies that make accessing contraception difficult or inaccessible
